# Comparing therapeutic outcomes: radioactive iodine therapy versus non-radioactive iodine therapy in differentiated thyroid cancer

**DOI:** 10.3389/fendo.2024.1442714

**Published:** 2024-09-20

**Authors:** Robert Aurelian Tiucă, Oana Mirela Tiucă, Raluca Monica Pop, Ionela Maria Paşcanu

**Affiliations:** ^1^ Doctoral School of Medicine and Pharmacy, George Emil Palade University of Medicine, Pharmacy, Science, and Technology of Targu Mures, Targu Mures, Romania; ^2^ Department of Endocrinology, George Emil Palade University of Medicine, Pharmacy, Science, and Technology of Targu Mures, Targu Mures, Romania; ^3^ Compartment of Endocrinology, Mures County Clinical Hospital, Targu Mures, Romania; ^4^ Department of Dermatology, George Emil Palade University of Medicine, Pharmacy, Science, and Technology of Targu Mures, Targu Mures, Romania; ^5^ Dermatology Clinic, Mures County Clinical Hospital, Targu Mures, Romania

**Keywords:** differentiated thyroid cancer, radioactive iodine therapy, biochemical control, therapy outcomes, personalized management

## Abstract

**Introduction:**

Radioactive iodine (RAI) has been utilized for nearly 80 years in treating both hyperthyroidism and thyroid cancer, and it continues to play a central role in the management of differentiated thyroid cancer (DTC) today. Recently, the use of RAI therapy for indolent, low-risk DTC has generated considerable debate. This case-control study evaluated the therapeutic response in DTC patients, comparing outcomes between those who received RAI therapy and those who did not.

**Methods:**

The study included individuals diagnosed with either indolent or aggressive histological types of DTC who either underwent RAI therapy or did not. For each patient, information regarding demographics (age, sex, background), clinical data, laboratory parameters, pathological exam, history of RAI therapy, thyroid ultrasound findings, and loco-regional or distant metastasis was extracted. All group comparisons were made using a two-sided test at an α level of 5%.

**Results:**

Out of 104 patients diagnosed with DTC, 76 met the inclusion criteria and were subsequently divided into two primary groups based on their history of RAI ablation. The majority of patients underwent RAI therapy (76.3%). Most patients had a good biochemical (68.4%, p = 0.246) and structural control (72.4%, p = 0.366), without a significant difference between the two groups. RAI therapy significantly protected against incomplete biochemical control in the overall population (p = 0.019) and in patients with histological indolent DTC (p = 0.030). Predictive factors for incomplete biochemical control included male sex (p = 0.008) and incomplete structural control (p = 0.002) across all patients, regardless of the histological type.

**Discussions:**

While RAI therapy has traditionally been used to manage DTC, our study found no significant difference in biochemical and structural responses between patients who received RAI therapy and those who did not. However, RAI therapy emerged as a protective factor against incomplete biochemical control, even in histological indolent DTC cases. These findings suggest that while RAI therapy may not be universally necessary, it could be beneficial in reducing the risk of biochemical recurrence in select patient subgroups, such as those with incomplete structural control or male patients. Thus, a personalized approach to RAI therapy, tailored to individual risk factors, may improve patient outcomes without overtreatment.

## 1 Introduction

Thyroid cancer stands as the most common endocrine malignancy, with escalating incidence rates across numerous nations over recent decades (1, 2). However, despite this rise in incidence, the worldwide mortality rates associated with thyroid cancer have remained stable at lower levels (2). The epidemiological picture of thyroid cancer strongly implies the presence of an overdiagnosis phenomenon, primarily due to increased ultrasound screening (2, 3).

The papillary and follicular histological types of thyroid cancer are widely recognized as the most common types, being referred to as differentiated thyroid cancer (DTC) (4, 5). Most DTC cases are diagnosed in asymptomatic patients and present a favorable prognosis. Active surveillance has been recently proposed as a management option for thyroid cancers less than 1 cm, whereas surgery with or without subsequent radioactive iodine (RAI) is typically applied in thyroid cancers exceeding 1 cm in size (6).

RAI has been used for nearly 80 years in the treatment of both hyperthyroidism and thyroid cancer, maintaining a central part in the management of DTC even nowadays (7, 8). RAI administration should follow the Martinique Principles, ensuring a goal-tailored approach defined as remnant ablation, adjuvant treatment, or treatment of known disease (9, 10). As outlined in the American Thyroid Association (ATA) 2015 guidelines, adjuvant therapy following total thyroidectomy is advised for ATA intermediate-risk DTC, a recommendation categorized as weak with low-quality evidence. Moreover, adjuvant treatment for ATA high-risk DTC is routinely recommended with strong, moderate-quality evidence. Initial adjuvant treatment with RAI typically involves activities ranging between 30 mCI and 150 mCI of 131I (10, 11).

On the other hand, according also to ATA 2015 guidelines, the utilization of RAI for remnant ablation in low-risk DTC cases following thyroidectomy is not routinely advocated. However, this recommendation is weak with low-quality evidence, except in cases of unifocal papillary microcarcinoma without other adverse features where the recommendation is considered strong with moderate-quality evidence (11). Nevertheless, there is still ongoing scientific debate over the use of RAI in low-risk DTC cases, with two trials assessing the non-inferiority of no RAI treatment versus low-dose RAI in low-risk DTC: ION and ESTIMABL2 (12, 13).

Considering the ongoing debate regarding the benefits of RAI adjuvant treatment in indolent DTC, this study aimed to evaluate the biochemical response to therapy of patients diagnosed with DTC within our institution, comparing the outcomes between those who received RAI therapy and those who did not.

## 2 Material and methods

### 2.1 Study population

This retrospective case-control study enrolled patients with DTC who received RAI ablation (case group) or not (control group) and were admitted to our institution between 2021-2023. The cohort comprised individuals whose earliest documented DTC diagnosis dates back to 1978, with the most recent diagnosis recorded in 2022. The inclusion criteria were patients older than 18 years old, having a pathological diagnosis of DTC, and for whom data regarding treatment and clinical outcomes were available. Patients lacking comprehensive data regarding the pathological exam, RAI ablation history, and biochemical and structural responses after total thyroidectomy, with or without adjuvant RAI ablation, were excluded from the analysis.

### 2.2 Data collection

The data was collected using the hospital’s electronic databases and the patients’ medical records. For each patient, information regarding demographics (age, sex, background), clinical data, laboratory parameters, pathological exam, history of RAI therapy, thyroid ultrasound findings, and loco-regional or distant metastasis was extracted.

Regarding clinical data, the following were collected on admission: body mass index (BMI), smoking status, family history of thyroid cancer, and personal history of other thyroid diseases. A BMI <25 kg/m2 was considered normal weight, while ≥25 kg/m2 defined excess weight. The following laboratory parameters were analyzed: the latest thyroid-stimulating hormone (TSH), thyroglobulin (Tg), and anti-thyroglobulin antibodies (TgAb).

The histological types were divided into two categories:

- Indolent histological types included classic papillary carcinoma, papillary microcarcinoma, follicular variant of papillary microcarcinoma, follicular carcinoma, and Warthin-like variant.
- Aggressive histological types included the tall-cell variant, the poorly differentiated component of follicular carcinoma, follicular carcinoma with an insular carcinoma component, oncocytic carcinoma, and the Hobnail variant.

### 2.3 Response to therapy

We considered biochemical incomplete response at the last evaluation to be a stimulated thyroglobulin (stTg) value >1 ng/ml and non-stimulated thyroglobulin (nstTg) value >0.2 ng/ml in patients who underwent total thyroidectomy and RAI ablation, stTg value >2 ng/ml or nstTg value >0.2 ng/ml in patients who underwent only total thyroidectomy, and nstTg value >30 ng/ml in patients who underwent isthmolobectomy. Based on the thyroid ultrasound findings, we considered an incomplete structural response to be the presence of hypoechoic or isoechoic residual tissue in the thyroid bed or the presence of laterocervical lymph nodes with suspicious characteristics or distant metastasis (11, 14).

### 2.4 Study outcome

The primary endpoint of our study was to assess whether histological indolent DTC cases that did not receive RAI had a worse outcome regarding the biochemical and structural response to therapy compared to histological indolent DTC cases that received RAI therapy. Secondly, we investigated whether the patient’s sex, background, smoking status, age at diagnosis, RAI therapy history, body mass index, structural control, or histological type influenced the biochemical response to therapy.

### 2.5 Statistical analysis

For data collection, Microsoft Excel (Microsoft Corporation, Redmond, Washington, United States) was used. The statistical analysis was performed using IBM SPSS Statistics for Windows, Version 25.0 (IBM Corp., Armonk, New York, United States), with the following statistical tests: Kolmogorov-Smirnov normality test, the Mann-Whitney test for central tendencies comparison, Chi-square, and variants for testing associations between categorical variables, logistic regression models with single and multiple predictors. All group comparisons were made using a two-sided test at an α level of 5%.

### 2.6 Ethical approval

The study was conducted in accordance with the Declaration of Helsinki and approved by the Ethics Committee of the University of Medicine, Pharmacy, Science, and Technology “George Emil Palade” of Târgu Mureş no. 2706/27.12.2023. Informed consent was obtained from all subjects involved in the study.

## 3 Results

### 3.1 Study population’s clinical profile

Out of 104 patients diagnosed with DTC, 76 met the inclusion criteria and were subsequently divided into two primary groups based on their history of RAI ablation: the case group consisting of 58 patients who received RAI therapy, and the control group consisting of 18 patients who did not undergo RAI therapy. The median follow-up period for all study population was 4.0 years (1.0-9.0). The mean age at diagnosis was 48.5 ± 13.4 years. The majority of patients were females (n = 65, 85.5%) and had an urban background (n = 48, 63.2%). Most patients had excess weight (n = 63, 82.9%), were non-smokers (n = 53, 69.7%), and had no prior history of thyroid disease before being diagnosed with DTC (n = 39, 51.3%). Total thyroidectomy was the main surgical intervention performed (n = 75, 98.7%). Pathological examination revealed that most patients had an indolent histological type of DTC (n = 66, 88.8%). Following total thyroidectomy, the majority of patients underwent RAI therapy (n = 58 vs 18, 76.3% vs 23.7%). The median follow-up period was 5.5 years (2.0-10.0) for the case group, respectively 1.5 years (1.0-5.0) for the control group. During the most recent evaluation, the majority of patients had a good biochemical (n = 52, 68.4%) and structural (n = 55, 72.4%) control, with only a small proportion presenting with metastasis (n = 4, 5.3%). Regarding the patients who developed metastasis during follow-up, most of them had an indolent histological type (n = 3, 75%) and all of them (n = 4, 100%) had received prior RAI therapy.

No statistically significant differences were identified between the two study groups regarding age, gender, background, BMI, smoking status, surgical intervention, histological type, biochemical and structural control following therapy, and metastasis. On the other hand, the control group had a significantly shorter period of follow-up and a more prevalent history of thyroid disease. **Table 1** illustrates the entire study population’s clinical characteristics and response to therapy.

**Table 1:** Clinical characteristics and response to therapy of the study population.

Variables	All patients (n = 76)	Case group* (n = 58)	Control group** (n = 18)	P value
Age at diagnosis (years)	48.5 ± 13.4	47.4 ± 13.6	52.1 ± 14.1	0.208
Follow-up period (years)	4.0 (1.0-9.0)	5.5 (2.0-10.0)	1.5 (1.0-5.0)	0.007
Gender				0.442
Male Female	11 (14.5%) 65 (85.5%)	10 (17.2%) 48 (82.8%)	1 (5.6%) 17 (94.4%)	
Background				0.786
Rural Urban	28 (36.8%) 48 (63.2%)	22 (37.9%) 36 (62.1%)	6 (33.3%) 12 (66.7%)	
BMI				0.172
Normal weight Excess weight	13 (17.1%) 63 (82.9%)	12 (20.7%) 46 (79.3%)	1 (5.6%) 17 (94.4%)	
Smoking status				0.559
Smoker Non-smoker	23 (30.3%) 53 (69.7%)	19 (32.8%) 39 (67.2%)	4 (22.2%) 14 (77.8%)	
Personal history of thyroid disease				0.033
Without Nodular goiter Hashimoto’s thyroiditis Nodules + thyroiditis	39 (51.3%) 26 (34.2%) 4 (5.3%) 7 (9.2%)	35 (60.3%) 16 (27.6%) 3 (5.2%) 4 (6.9%)	4 (22.2%) 10 (55.6%) 1 (5.6%) 3 (16.7%)	
Surgical intervention				0.236
Total thyroidectomy Isthmolobectomy	75 (98.7%) 1 (1.3%)	58 (100%) 0 (0%)	17 (94.4%) 1 (5.6%)	
Histological type				0.105
Indolent Aggressive	66 (88.8%) 10 (13.2%)	48 (82.8%) 10 (17.2%)	18 (100%) 0 (0%)	
Biochemical control at the last evaluation				0.246
Good response Incomplete response	52 (68.4%) 24 (31.6%)	42 (72.4%) 16 (27.6%)	10 (55.6%) 8 (44.4%)	
Structural control at the last evaluation				0.366
Good response Incomplete response	55 (72.4%) 21 (27.6%)	40 (69.0%) 18 (31.0%)	15 (83.3%) 3 (16.7%)	
Metastasis following therapy				0.567
Present Absent	4 (5.3%) 72 (94.7%)	4 (6.9%) 54 (93.1%)	0 (0%) 18 (100%)	

BMI, body mass index.

*DTC cases that received RAI therapy.

**DTC cases that did not receive RAI therapy.

#### 3.1.1 Predictive factors for incomplete biochemical control for all study population

Subsequently, logistic regression was performed, testing the predictive power of gender, follow-up period, background, smoking status, age at diagnosis, RAI therapy, histological type, excess weight, and structural control on the biochemical control. As shown in **Table 2**, RAI therapy and the follow-up period were significant protective factors. At the same time, male sex and incomplete structural control significantly predicted an incomplete biochemical control, with a Nagelkerke R square of 0.478, showing a moderate fit.

**Table 2:** Predictive factors for incomplete biochemical control for all study population.

Variable	OR	95% CI	P value
Male sex	16.601	2.07-132.70	0.008
Follow-up period	0.827	0.68-0.99	0.041
Urban background	0.414	0.10-1.64	0.210
Smoking	2.510	0.61-10.26	0.200
Age at diagnosis	0.985	0.93-1.03	0.558
RAI therapy	0.108	0.01-0.69	0.019
Aggressive histological type	1.892	0.28-12.48	0.508
Excess weight	0.220	0.03-1.46	0.118
Incomplete structural control	13.088	2.47-69.18	0.002

OR, odds ratio; 95% CI, 95% confidence interval; RAI, radioactive iodine.

### 3.2 Histological indolent DTC cases

Furthermore, histological indolent DTC cases (n = 66) were divided into two additional groups based on their history of RAI therapy: the case group consisting of patients with histological indolent DTC who received RAI therapy, and the control group consisting of patients with histological indolent DTC who did not undergo RAI therapy. Most patients received RAI therapy (n = 48 vs 18, 72.7% vs 27.3%). The majority of patients were female, non-smokers, and had good biochemical and structural control. Most patients who received RAI therapy had no prior history of thyroid disease (n = 27, 56.3%), while a history of nodular goiter was the most prevalent in patients who did not receive RAI therapy (n = 10, 55.6%). No statistically significant differences were identified between the two groups regarding gender, BMI, smoking status, personal history of thyroid disease, and biochemical and structural control, with the only significant difference being the follow-up period (**Table 3**).

**Table 3:** Comparison between histological indolent DTC cases.

Variable	Case group* (n = 48)	Control group** (n = 18)	P-value
Follow-up period (years)	6.5 (3.0-10)	1.5 (1.0-5.0)	0.003
Gender			0.430
Female Male	41 (85.4%) 7 (14.6%)	17 (94.4%) 1 (5.6%)	
BMI			0.156
Normal weight Excess weight	11 (22.9%) 37 (77.1%)	1 (5.6%) 17 (94.4%)	
Smoking status			0.383
Smoker Non-smoker	17 (35.4%) 31 (64.6%)	4 (22.2%) 14 (77.8%)	
Personal history of thyroid disease			0.091
Without Nodular goiter Hashimoto’s thyroiditis Nodules + thyroiditis	27 (56.3%) 15 (31.2%) 2 (4.2%) 4 (8.3%)	4 (22.2%) 10 (55.6%) 1 (5.6%) 3 (16.7%)	
Biochemical control			0.144
Good response Incomplete response	36 (75.0%) 12 (25.0%)	10 (55.6%) 8 (44.4%)	
Structural control			0.354
Good response Incomplete response	33 (68.8%) 15 (31.2%)	15 (83.3%) 3 (16.7%)	

DTC, differentiated thyroid cancer; BMI, body mass index.

*histological indolent DTC cases that received RAI therapy.

**histological indolent DTC cases that did not receive RAI therapy.

#### 3.2.1 Predictive factors for incomplete biochemical control for histological indolent DTC cases

Logistic regression was performed, testing the predictive power of gender, follow-up period, background, smoking status, age at diagnosis, RAI therapy, excess weight, and structural control on the biochemical control. Male sex and incomplete structural control significantly predicted an incomplete biochemical control, while RAI therapy was a significant protective factor (**Table 4**), with the model showing a good fit (Nagelkerke R square 0.537).

**Table 4:** Predictive factors for incomplete biochemical control for histological indolent DTC patients.

Variable	OR	95% CI	P value
Male sex	17.363	1.19-251.66	0.036
Follow-up period	0.796	0.62-1.01	0.070
Urban background	0.459	0.09-2.26	0.340
Smoking	2.720	0.44-16.55	0.277
Age at diagnosis	0.973	0.90-1.04	0.436
RAI therapy	0.090	0.01-0.79	0.030
Excess weight	0.169	0.01-1.97	0.156
Incomplete structural control	15.656	2.00-122.36	0.009

DTC, differentiated thyroid cancer; OR, odds ratio; 95% CI, 95% confidence interval; RAI, radioactive iodine.

## 4 Discussions

In this study evaluating the biochemical and structural responses to therapy in patients with DTC, we observed favorable outcomes across the cohort, with 68.4% of patients demonstrating good biochemical response and 72.4% showing good structural response. Notably, our analysis revealed no significant difference in outcomes between patients who received RAI and those who did not. Our study included predominantly patients with histological indolent DTC, the majority of whom underwent total thyroidectomy and RAI therapy. Only one patient underwent isthmolobectomy, having an indolent histological type, without subsequent completion of thyroidectomy or RAI therapy. The predominance of total thyroidectomy and RAI therapy in our study population may be explained by the fact that several patients were treated according to different guidelines, considering that the earliest DTC diagnosis dates back to 1978 and the latest to 2022. Over the recent decades, through careful analysis of patient outcomes, it has been observed that most DTC cases present a low risk of recurrence following total thyroidectomy. This observation has led to significant changes in the management strategies for DTC, influencing the selection of surgical intervention type and the utilization of RAI therapy (15, 16).

With the introduction of the ATA guidelines in 2015, lobectomy started to become an effective and safe initial treatment of DTC tumors measuring not only < 1 cm but also for intrathyroidal tumors between 1 and 4 cm, having an overall lower risk of complications without compromising the patient’s oncological outcome (11, 16–18). However, the decision of initial surgical intervention should not be based solely on tumor size but must also consider all other risk factors. Moreover, even without associated particular risk factors, tumors between 2 and 4 cm have an increased risk of local recurrence (19). Therefore, informing the patient regarding this aspect might change the decision of the initial surgical intervention based on the patient’s preference. On the other hand, recent trends show a preference towards lobectomy even in low-risk DTC with extrathyroidal extension, despite a slight possibility of undertreating patients with high-risk features (20). Furthermore, active surveillance has been shown to achieve outcomes comparable to immediate surgery for small, low-risk DTC. However, the success of active surveillance depends on substantial patient compliance and clinician diligence, as it requires regular monitoring and a proactive approach (21–23).

RAI therapy has been effectively used over the past several decades to treat various thyroid disorders, including DTC. Over time, the indications for RAI therapy in DTC have changed, and current recommendations advocate for a more risk-tailored approach to its utilization (15). Similar to the de-escalation seen with total thyroidectomy, recent studies showed that RAI therapy has been used less frequently after the implementation of ATA 2015 guidelines (24–26).

Our study found that most patients presented a good biochemical (68.4%) and structural response (72.4%), regardless of RAI therapy history. Moreover, we found no significant difference between the biochemical (p = 0.246) or structural (p = 0.366) response between the two study groups. Considering that most patients had an indolent histological type (88.8%), our results are similar to other studies found in the literature conducted on low-risk DTC. A recent study on more than 2000 patients diagnosed with low-risk DTC found that RAI ablation did not have a significant survival advantage compared to no-RAI therapy, having similar recurrence rates (27). In 2022, the results from the ESTIMABL2 clinical trial were published after three years of follow-up strategy in patients with low-risk DTC treated only with thyroidectomy, without RAI therapy. The majority of enrolled patients were female (83%) with papillary thyroid carcinoma (96%). At three years post-randomization, the recurrence-free survival was 95.6% for the no-RAI group vs 95.9% for the RAI group, meeting the non-inferiority criteria (13). Our study had 4 patients who developed metastasis during follow-up. Most patients had an indolent histological type (n = 3, 4.5%) and all patients underwent RAI therapy prior to metastasis occurrence. The overall higher sample size of patients with indolent histological type versus aggressive histological type might explain the higher proportion of histological indolent DTC among the patients with metastasis. It is possible that the indolent histological type in these cases did not fully reflect the aggressive potential of the tumors, especially if aggressive mutations such as BRAFV600E or pTERT were present. Furthermore, subclinical metastatic foci could have been present and not detected during initial evaluations. Nevertheless, the development of metastasis in the context of prior RAI therapy suggests that while RAI may reduce recurrence risk, it does not eliminate the possibility of distant metastasis.

Pasqual et al. findings suggest that at least one-fifth of low-risk DTC patients may be overtreated with RAI therapy (26). Considering this observation and our study results regarding response to therapy, which showed no difference between the RAI therapy group and the no-RAI therapy group, regardless of histological type, there is a possibility that several patients included in our analysis were overtreated with RAI therapy. RAI therapy overtreatment might lead not only to an increased risk of complications such as taste and smell impairment, sialadenitis, and increased risk of secondary malignancy but also to increased costs of healthcare (28, 29). Using a low-activity RAI might reduce the associated complications with RAI therapy without negatively influencing the patient’s outcome. A meta-analysis published in 2021 by James et al. showed that low-activity RAI has similar efficacy, but reduced morbidity compared to high-activity RAI in low and intermediate-risk DTC (30).

Potential risk factors for thyroid cancer might be classified as high risk, such as radiation exposure, genetic alterations, or hereditary disorders, and low risk, like iodine deficiency, autoimmunity, thyroid nodules, high BMI, high TSH, and lifestyle (31). In our study population, most patients had no thyroid disease before thyroid cancer diagnosis. However, there was a significant difference between the two study groups regarding the personal history of prior thyroid diseases (p = 0.033). When looking at the two groups separately, most patients in the RAI therapy group had no prior history of thyroid diseases (60.3%). In contrast, the majority of patients in the no-RAI therapy group had a history of nodular goiter (55.6%). This finding might be explained by the low number of patients included in the no-RAI therapy group.

As stated earlier, lifestyle is considered a low-risk factor for thyroid cancer. Most patients included in our study were non-smokers (69.7%). Although smoking has been shown to increase the risk of several cancers in thyroid cancer, it seems it has a protective effect, reducing the risk of both papillary thyroid cancer as well as follicular thyroid cancer (32). This might be explained by the TSH-lowering effect and anti-estrogenic effects of smoking, resulting in a reduced thyroid cancer risk (31, 33). We found that most patients had excess weight (82.9%), although the BMI was calculated based on height and weight at the last evaluation. Therefore, no assumptions can be made regarding the BMI at DTC diagnosis. Obesity is associated with insulin resistance, leading to increased binding of insulin-like growth factor1 (IGF-1) to its receptor, acting like a growth factor and therefore promoting oncogenesis, tumor progression, and metastasis (31, 34).

We found no significant difference between the two study groups regarding the biochemical control at the last evaluation (p = 0.246), with most patients having a good biochemical control (68.4%). Moreover, similar results were found when comparing the biochemical control between indolent histological types with or without RAI therapy (p = 0.144). These results suggest that RAI therapy was not a definitive factor in achieving good biochemical control, similar to other studies (13, 27). However, when considering all study population, we found RAI therapy (OR = 0.108, 95% CI = 0.01-0.69, p = 0.019) and follow-up duration (OR = 0.827, 95% CI = 0.68-0.99, p = 0.041) to be protective factors against an incomplete biochemical control. Good biochemical control observed in a short follow-up period could be explained by the immediate and effective response to aggressive initial treatments and by the slow progression of the disease. However, a brief period of follow-up has its pitfalls. While short follow-up periods may show initially positive outcomes, they risk missing late recurrences. Longer monitoring periods are required to detect stable trends and confirm the absence of residual or recurrent disease. Thus, extended follow-up is necessary to assess biochemical control and ensure proper evaluation accurately. On the other hand, an incomplete structural control (OR = 13.088, 95% CI = 2.47-69.18, p = 0.002) and male sex (OR = 16.601, 95% CI = 2.07-132.70, p = 0.008) were found to be significant predictive factors for incomplete biochemical control. Similar results were found when looking at low-risk histological types, with RAI therapy history being a protective factor (OR = 0.090, 95% CI = 0.01-0.79, p = 0.030), while an incomplete structural control (OR = 15.656, 95% CI = 2.00-122.36, p = 0.009) and male sex (OR = 17.363, 95% CI = 1.19-251.66, p = 0.036) were predictive factors for an incomplete biochemical control. These results confirm similar findings in the literature (35–38). Thus, RAI therapy post-thyroidectomy, even in histological indolent DTC cases, might be necessary to achieve good biochemical control, especially in male patients. Therefore, a personalized approach in deciding to administrate RAI therapy is mandatory for each patient and should not be based solely on tumor size or histological type.

Good structural control was found in most patients (72.4%), with no significant difference between the two studied groups (p = 0.366). Similarly, structural control was adequate for most patients with indolent histological types with or without RAI therapy, with no significant difference between the two cohorts (p = 0.354). Considering the retrospective nature of our study, predictive factors for incomplete structural control have not been assessed. The definition of incomplete structural control currently needs to be improved, and determining predictive factors in a retrospective study might be subject to biased interpretation of previous thyroid ultrasound results. ATA defines incomplete structural response as the presence of persistent or newly diagnosed loco-regional or distant metastasis, expanding the definition to indeterminate for any structural findings that cannot be classified as benign or malignant (11). Therefore, determining an incomplete structural response might be challenging when it is based solely on thyroid ultrasound. The clinician’s experience has to be one of the most critical factors that influence classifying a patient as having an incomplete structural control, especially when the biochemical control is excellent. Moreover, it might be beneficial to have the same physician perform the thyroid ultrasound consistently to reduce misclassification. Common benign conditions that may mimic incomplete structural control include postoperative scars, suture granuloma, reactive lymphoid hyperplasia, or remaining thyroid tissue (39). Thyroid bed nodules are a common ultrasound finding after total thyroidectomy and may mimic an incomplete structural control. However, thyroid bed nodules usually fail to become clinically significant and should not be aggressively investigated unless they develop suspicious sonographic features, associate suspicious cervical lymph nodes or rising Tg (40). A recent study that investigated more than 3000 thyroid bed lesions found that only the presence of punctate echogenicity or the history of positive lymph nodes at thyroidectomy were associated with malignancy, concluding that lesions under 6 mm without these associated risk factors had minimal risk of malignancy (41). Therefore, determining an incomplete structural response might be challenging when it is based solely on previous thyroid ultrasound results, without directly following the patient, making it a difficult variable to assess in a retrospective study. Future prospective studies should focus on clearly defining incomplete structural control in DTC, reducing the confusing interpretations of thyroid ultrasounds.

Future research that might result in more personalized management should include molecular biology assessment of gene mutations associated with unfavorable prognoses. For instance, the co-existence of telomerase reverse transcriptase promoter (pTERT) and B-Raf Proto-Oncogene, Serine/Threonine Kinase (BRAF) V600E has been associated with increased risk of recurrence, distant metastasis, and mortality (42, 43). Moreover, determining genetic polymorphisms in numerous genes, such as Rearranged during Transfection (RET), B-cell lymphoma 2-associated X protein (BAX), tumor protein 53 (TP53), and nitric oxide synthase 3 (NOS3), might also help towards a more risk-tailored management in DTC cases. The authors previously conducted a thorough review of several genetic variants and their implications in DTC (44). This genetic information could result in a more objective decision regarding the extension of initial surgical intervention, subsequent RAI therapy, and future follow-up management.

Our study had several limitations. First, given its retrospective nature, all data were collected from medical records, and no additional missing data could be obtained. Lack of complete information regarding the histological examination, pre-operative Tg, presence of lymph node metastasis, history of RAI therapy, and total RAI dose were drawbacks that excluded several patients from our analysis, reducing the final study population. Future similar research might benefit from a prospective enrollment of patients. Secondly, another limitation was the small sample of DTC cases that did not undergo RAI therapy. However, after 2015, the utilization of RAI therapy has decreased for indolent cases. Therefore, we can expect fewer low-risk DTC cases that will receive RAI therapy. Nevertheless, it would be useful to investigate the benefits of RAI therapy in histological indolent DTC cases in more extensive cohort studies, considering also the genetic implications mentioned earlier. The short period of follow-up could be another limitation of our study. This can result in an overestimation of initial treatment efficacy, thereby skewing the study results and leading to potentially inaccurate conclusions about the long-term effectiveness of the therapeutic interventions. Finally, potential discrepancies surrounding the ultrasound diagnosis criteria of structural control limit the conclusions that can be drawn from our analysis, as predictive factors for incomplete structural control were not determined.

## 5 Conclusions

Recently, there has been a notable shift towards a more individualized approach to managing DTC, following decades characterized by largely uniform treatment and monitoring guidelines. Several risk factors should be considered when deciding on the initial surgical intervention or additional RAI therapy. Based on this study’s results, RAI therapy may be beneficial in the long term in all histological types of DTC, reducing the risk of biochemical incomplete response and, consequently, the risk of recurrence, especially in male patients. Therefore, incorporating RAI therapy into the treatment plan for DTC patients in a personalized approach, tailored to individual risk factors, may improve the overall prognosis and long-term survival rates, without overtreatment.

## Data Availability

The raw data supporting the conclusions of this article will be made available by the authors, without undue reservation.
